# Atrial Fibrillation in Advanced CKD: To Anticoagulate, or Not, and With What?

**DOI:** 10.34067/KID.0000000869

**Published:** 2025-09-25

**Authors:** Muhammad S. Ajmal, Wolfgang C. Winkelmayer

**Affiliations:** Section of Nephrology, Baylor College of Medicine, Houston, Texas

**Keywords:** cardiovascular disease, CKD

Atrial fibrillation (AF) is the most common arrhythmia. Its incidence and prevalence have been increasing in the United States and globally, and AF has been projected to affect approximately 30 million Americans in 2030. CKD predisposes to developing AF; both lower eGFR and higher albuminuria are associated with the risk of incident AF.^1^ Furthermore, CKD is also an independent risk factor for ischemic stroke,^1^ a dreaded complication of AF. Luckily, interventions are available to reduce the risk of stroke in patients with AF. Oral anticoagulation is among the most effective interventions in modern medicine. In a meta-analysis of pivotal trials, adjusted dose warfarin reduced stroke risk by 62% (95% confidence interval [CI], 48% to 72%) and all-cause mortality by 26% (95% CI, 4% to 43%) compared with placebo.^2^ These benefits far outweighed the smaller increase in bleeding risk in the warfarin groups. Very limited trial evidence supporting the efficacy and safety of warfarin specifically in persons with mostly moderate CKD is available.^3^ Clinical use of warfarin is limited by a markedly reduced time in the therapeutic range in patients with severe CKD and its association with calciphylaxis.

More recently, the registrational trials for direct-target oral anticoagulants (DOACs), which do not require therapeutic monitoring, demonstrated noninferiority compared with warfarin for stroke reduction and bleeding risk, including in patients with CKD down to a creatinine clearance of 30 ml/min (25 ml/min for apixaban), which were the exclusion thresholds for kidney function in these pivotal trials.^4^ Thus, both the original placebo-controlled trials for warfarin as well as the subsequent registrational trials for DOACs systematically excluded patients with advanced kidney disease, which has left an important evidence gap. As a result, the available evidence on the use of anticoagulation in patients with AF and stage 4 of 5 CKD (nondialysis) is largely observational.

The report by Lam *et al.*^5^ in this issue of *Kidney360* attempts to fill part of this evidence gap through a retrospective, observational study comparing the effectiveness and safety of apixaban with warfarin in patients with AF across the spectrum of non–dialysis-dependent CKD patients in real-world settings in Australia and Canada. Five retrospective cohorts that included 106,102 adults with AF, a new dispensation of apixaban or warfarin, and a recorded eGFR, which was grouped as ≥60, 45–59, 30–44, and <30 ml/min per 1.73 m^2^. Exposure propensity score matching generated cohorts of 19,299 apixaban and 19,299 warfarin users whose observed baseline characteristics were comparable, thus limiting potential confounding by (contra)indication from observed factors. Within this cohort, 3290 (8.5%) participants had CKD Stage 4 or 5.

More than 90% of patients had CHA_2_DS_2_-VASc scores of 2 or more, while two thirds of participants had HAS-BLED scores of 2 or less in both warfarin and apixaban groups. Although the HAS-BLED scores were relatively low, potentially indicating lower bleeding risk, one needs to consider that not all variables that factor into this bleeding score could be ascertained in the databases from which the cohorts were derived. Another potential limitation was that international normalized ratio measurements were not available, and thus, the investigators were unable to report time in therapeutic range among warfarin users.

The effectiveness outcome was time to the earlier between hospitalization for ischemic stroke or transient ischemic attack and death from any cause, and the main safety outcome was time to the earliest among hospitalizations for intracranial, upper or lower gastrointestinal, or other bleeding. Follow-up was ended at 1 year from the cohort entry date.

Over the 1-year follow-up, the composite effectiveness outcome occurred in 4130 (10.7%) patients, of which 89.5% were deaths and only 433 (10.5%) of these events were stroke or transient ischemic attack. The composite bleeding outcome occurred in 717 (1.8%) patients, of which almost two thirds were gastrointestinal bleeding episodes (*N*=452); intracranial bleeds were relatively rare (*N*=105).

For earlier stages of CKD or for people without CKD (based on their eGFR alone), the results using these real-world data mirrored the findings from the pivotal apixaban trial for stroke prevention in AF. That trial, ARISTOTLE,^6^ was a randomized, double-blind noninferiority trial which showed that apixaban was not only noninferior, but superior to warfarin in preventing stroke or systemic embolism, caused less bleeding, and resulted in lower mortality. Exclusion criteria included severe CKD (serum creatinine level >2.5 mg/dl or calculated creatinine clearance <25 ml/min); 15.1% of participants had eGFR 30–50 ml/min range and 1.5% had eGFR <30 ml/min (*N*=269). The beneficial effects of apixaban versus warfarin on the rates of stroke or systemic embolism and major bleeding were subsequently shown to be consistent in patients with normal, moderately reduced, or poor kidney function as well as among those with worsening kidney function during follow-up.^7^

Although the study by Lam *et al.* had limited sample size and power within the stratum with advanced CKD (stages 4 and 5), they were able to demonstrate a reduction in bleeding risk with apixaban compared with warfarin, hazard ratio (HR), 0.68 (95% CI, 0.47 to 0.99). However, the association with the ischemic events (which included mortality from any cause) was near unity with wide confidence limits (HR, 0.99; 95% CI, 0.68 to 1.45). Because there were relatively few patients with eGFR <15 ml/min per 1.73 m^2^, no conclusion regarding effectiveness, safety, or net benefit could be drawn for that category alone. The mean eGFR within the CKD stages 4 and 5 category was 25.0 and 21.7 ml/min in the matched apixaban and warfarin groups, which is the eGFR range below which the benefits and risks of apixaban are most uncertain and previous literature had limited data. Thus, this report adds meaningful safety data for the selection of an oral anticoagulant in patients with CKD 4 or 5.

This new study complements two other recently published studies using data from the United States that were able to shed brighter light on this question. Identifying patients with CKD stage 4 or 5 from diagnosis codes in Medicare fee-for-service claims rather than eGFR from creatinine measurements, Fu *et al.*^8^ demonstrated that bleeding was almost double in warfarin users versus apixaban users (HR, 1.85; 95% CI, 1.59 to 2.15), while the comparative effectiveness was also near unity: the rates of all-cause mortality and ischemic stroke were similar for warfarin versus the referent, apixaban (HR, 1.08; 95% CI, 0.98 to 1.18 and HR, 1.14; 95% CI, 0.83 to 1.57, respectively). Similarly, Xu *et al.*^9^ used the data of a large national insurer and included adults with AF and CKD stage 4 or 5 (mean eGFR 24.7 ml/min) who initiated anticoagulant treatment. Compared with warfarin, apixaban was associated with almost half the risk of major bleeding (incidence rate, 1.5 versus 2.9 per 100 person-years; HR, 0.53; 95% CI, 0.39 to 0.70) and similar risks for stroke/systemic embolism (HR, 0.80; 95% CI, 0.59 to 1.09) and death (HR, 1.03; 95% CI, 0.82 to 1.29).

Current cardiology clinical practice guidelines^10^ have a class 1 recommendation for using warfarin or preferably evidence-based doses of direct thrombin or factor Xa inhibitors in patients with CKD 3 and AF with elevated stroke risk. Recommendations become less confident for CKD stage 4 (class 2A) and for CKD stage 5 and patients on dialysis (class 2B), suggesting treatment may be reasonable with warfarin or labeled doses of DOACs (in CKD stage 4) or apixaban (in CKD stage 5) to reduce the risk of stroke. The 2024 update of the Kidney Disease Improving Global Outcomes CKD guidelines is more specific and recommends the use of non–vitamin K antagonist oral anticoagulants in preference to vitamin K antagonists (*e.g*., warfarin) for thromboprophylaxis in AF in people with CKD stages 1–4 (level of evidence, 1C, low).^11^

These three recent observational studies including the one in this issue of *Kidney360* by Lam *et al.* should direct physicians who, following a shared decision-making process with their patients, wish to initiate anticoagulation in the setting of AF and severe CKD to favor apixaban over warfarin. However, neither of these studies inform the more relevant, preceding clinical question, which is whether patients with advanced CKD should be anticoagulated at all given that the net benefit of anticoagulation versus not using any anticoagulation remains unknown in advanced CKD where not only thromboembolic but also bleeding risks are high. Conclusively resolving this clinical conundrum would require placebo-controlled trials.
